# Harmonizing variant classification for return of results in the All of Us Research Program

**DOI:** 10.1002/humu.24317

**Published:** 2021-12-28

**Authors:** Steven M. Harrison, Christina A. Austin‐Tse, Serra Kim, Matthew Lebo, Annette Leon, David Murdock, Aparna Radhakrishnan, Brian H. Shirts, Marcie Steeves, Eric Venner, Richard A. Gibbs, Gail P. Jarvik, Heidi L. Rehm

**Affiliations:** ^1^ Broad Institute of MIT and Harvard Cambridge Massachusetts USA; ^2^ Laboratory for Molecular Medicine Mass General Brigham Boston Massachusetts USA; ^3^ Center for Genomic Medicine Massachusetts General Hospital Boston Massachusetts USA; ^4^ Color Health Burlingame California USA; ^5^ Human Genome Sequencing Center Baylor College of Medicine Houston Texas USA; ^6^ University of Washington Medical Center Seattle Washington USA

**Keywords:** All of Us Research Program, data sharing, variant classification

## Abstract

The All of Us Research Program (AoURP) is a historic effort to accelerate research and improve healthcare by generating and collating data from one million people in the United States. Participants will have the option to receive results from their genome analysis, including actionable findings in 59 gene‐disorder pairs for which disorder‐associated variants are recommended for return by the American College of Medical Genetics and Genomics. To ensure consistent reporting across the AoURP, in a prelaunch study the four participating clinical laboratories shared all variant classifications in the 59 genes of interest from their internal databases. Of the 11,813 unique variants classified by at least two of the four laboratories, classifications were concordant with regard to reportability for 99.1% (11,711), with only 0.9% (102) having reportability differences. Through variant reassessment, data sharing, and discussion of rationale, participating laboratories resolved all 102 reportable differences. These approaches will be maintained during routine AoU reporting to ensure continuous classification harmonization and consistent reporting within AoURP.

## 1 INTRODUCTION

The All of Us Research Program (AoURP), sponsored by the National Institutes of Health (NIH), is a historic effort to enroll a diverse group of at least one million individuals in the United States to accelerate biomedical research and improve health (All of Us Research Program Investigators et al., 2019). The AoURP aims to achieve this goal by building a comprehensive research resource composed of surveys, biometrics, genetics, and electronic health records from enrolled participants and making these data available for research exploring biological, social, and environmental determinants of health and disease. Participants in the AoURP can elect to receive two classes of health‐related reports based on their genetic findings: (1) a Medicine and Your DNA report and (2) a Hereditary Disease Risk (HDR) report. The Medicine and Your DNA report is based on analysis of pharmacogenomic alleles in seven genes with known gene–drug interactions, in accordance with guidance from the Clinical Pharmacogenetics Implementation Consortium (Relling & Klein, 2011). The HDR report is based on analysis of genetic variants known to cause disorders for which there are established interventions, in accordance with recommendations of the American College of Medical Genetics and Genomics (ACMG) for reporting of secondary findings related to 59 genes, version 2.0 (Kalia et al., 2017). Recently, version 3.0 of the ACMG list was released (Miller et al., 2021) and the added content will be considered for return in the future, pending regulatory approval.

To generate genomic data from biosamples contributed by participants, AoURP funded three Genome Centers for whole genome sequencing: Baylor College of Medicine in partnership with Johns Hopkins University (BCM/JHU), Broad Institute of MIT and Harvard in partnership with Color Health and the Mass General Brigham (MGB) Laboratory for Molecular Medicine (Broad/Color/LMM), and Northwest Genomics Center at the University of Washington (UW). Each Genome Center supports genome sequencing (BCM, Broad, and UW), genotype arrays (JHU, Broad, and UW), and has a clinical validation laboratory (CVL) for orthogonal confirmation and interpretation (BCM, Color, and UW). After genomic data are generated at one of the Genome Centers, data from participants consented for return of genomic results are shared with the CVLs. Each CVL will perform pharmacogenomics analysis and variant classification of any variants in the HDR genes followed by orthogonal confirmation of pathogenic (P) or likely pathogenic (LP) variants. Classified variants will then flow into the AoU Report and Harmonization Platform where reports are signed out pending resolution of any conflicts in variant classification that may exist across the three CVLs. Reports will then be delivered to participants through a participant portal in conjunction with support provided by the AoU Genetic Counseling Resource.

Because the United States Food and Drug Administration (FDA) determined that the AoURP return of results proposed plan met criteria for a Significant Risk (SR) Device Study, an investigational device exemption (IDE) was deemed required for the AoURP. Genome centers collaborated to produce standard procedures for whole genome sequencing, variant calling, data interpretation, and return‐of‐results to ensure genomic data generated for any given AoURP participant would be accurate and equivalent across genome centers (Venner et al., 2021). The accuracy and precision of whole genome sequencing as a device for the return of certain health‐related genomic results was determined to be sufficient, and an IDE was granted by the FDA for the AoURP.

In addition to harmonization of whole genome sequencing and variant calling procedures across genome centers, harmonization of variant classification is also critical to ensure consistency of results being returned to AoURP participants. Differences in variant classification can result in individuals with the same variant having different medical management recommendations and follow‐up. Variant classification differences can occur for a multitude of reasons, such as differences in date of assessment, availability of internal data, and subjectivity in weighting evidence types; however, studies have shown that sharing data and discussion of rationale can increase variant classification concordance (Amendola et al., 2020; Harrison et al., 2017, 2018; Lebo et al., 2018). The AoURP decided to only return variants classified as P or LP, which is in accordance with the 2015 ACMG/Association of Molecular Pathology (ACMG/AMP) guideline for germline sequence variant classification and is consistent with current practices in the field (eMERGE Clinical Annotation Working Group, 2020; Hart et al., 2019; Richards et al., 2015) and updated recommendations released as part of the ACMG version 3.0 list (Miller et al., 2021).

To ensure consistent reporting between CVLs, in a prelaunch study the three CVLs, plus the MGB LMM, which coordinates the variant harmonization process, shared all variant classifications in the 59 genes of interest from their internal databases. Laboratories collaborated to identify and resolve any reportable differences in variant classifications before initiating reporting to AoU participants.

## 2 METHODS

In July 2019, the three CVLs and LMM shared all classified sequence variants in the 59 genes of interest from their internal databases. Additional annotations were shared from each laboratory when available, such as date of classification, disorder against which pathogenicity was asserted, and number of observations. To facilitate accurate comparison of shared variants and normalize variant expressions, the ClinGen Allele Registry was utilized to provide a canonical allele identifier for each variant (Pawliczek et al., 2018). For variant reassessment, the assigned laboratory was provided with the classification(s) from other participating laboratories, along with any additional annotations. For variants that remained discrepant after reassessment, the laboratory(ies) performing subsequent reassessments were also provided with classification notes and rationale from laboratory(ies) that had performed the prior reassessment. Variants were classified using the 2015 ACMG/AMP guidelines as well as additional guidance from the ClinGen Sequence Variant Interpretation working group and gene‐centric specifications from expert panels when available (https://clinicalgenome.org/svi/). Discussion regarding classification rationale and weighting of evidence occurred via email or were presented on AoU Clinical Interpretation and Reporting Working Group calls, which included representation from each CVL and LMM.

## 3 RESULTS

### 3.1 Shared variant classification analysis

**Figure 1:**
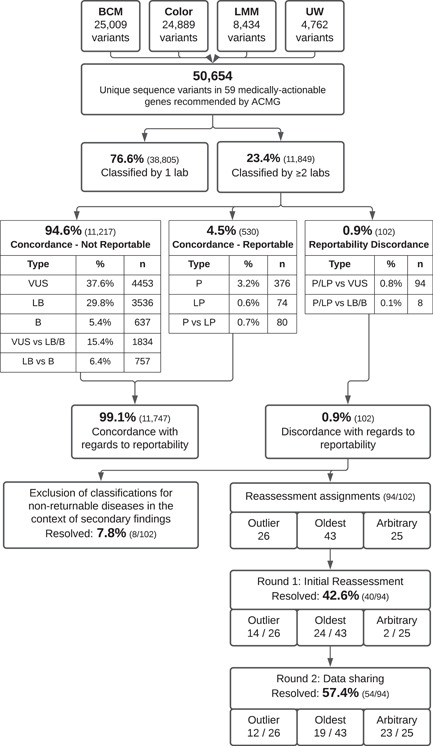
Variant classification harmonization process in the AoURP. AoURP, All of Us Research Program

Between the three CVLs and LMM, 50,654 unique sequence variants in the 59 genes on version 2 of the ACMG secondary finding list were identified, of which 23.4% (11,849 variants) were classified by at least two of the laboratories (Figure 1). As only variants classified as P and LP will be reported and returned to AoURP participants, shared variant classifications were then compared with determine concordance with regard to reportability (P/LP vs. VUS/LB/B). Across the 11,849 shared variants, 94.6% (11,217 variants) had concordant nonreportable classifications, which included variants concordantly classified as uncertain significance (VUS; 37.6%, 4453 variants), likely benign (LB; 29.8%, 3536 variants), or benign (B; 5.4%, 637 variants), as well as variants with VUS versus LB/B conflicting classifications (15.4%, 1834 variants) and LB versus B conflicting classifications (6.4%, 757 variants) as these variant classifications are still concordant with regard to nonreportability. Additionally, 4.5% (530 variants) had classifications that were concordant with respect to being reportable, including 3.2% (376 variants) with concordant P classifications, 0.6% (74 variants) with concordant LP classifications, and 0.7% (80 variants) with P versus LP classification differences. Overall, with regard to AoURP variant reportability, classifications were concordant for 99.1% (11,747) of shared variants, with only 0.9% (102 variants) having differences in reportability (i.e. P/LP vs. VUS/LB/B). Of the 102 variants with reportability differences (Tables 1 and S1), the most common conflict type was VUS versus LP (76.5%; 78 variants), followed by VUS versus P (15.7%; 16 variants).

**Table 1:** Classification difference categories for potentially reportable variants and outcomes of resolution process

Starting conflict type (*n*)	Resolved classification (*n*)
LB versus LP (6)	LP (2)
	VUS (3)
	NR (1)
LB versus P (2)	P (1)
	LP (1)
VUS versus LP (78)	LP (31)
	VUS (41)
	NR (6)
VUS versus P (16)	P (3)
	LP (6)
	VUS (4)
	NR (3)
LP to P (80)^a^	P (42)
	LP (2)

Abbreviations: LB, likely benign; LP, likely pathogenic; P, pathogenic; VUS, variants of uncertain significance.

^a^ LP to P resolution still in process; NR, not reportable (classification difference persists but for different diseases, one of which is not returnable in the context of secondary findings).

Missense variation was the most common variant type (49.1%; 5821 variants) across the 11,849 variants classified by at least two laboratories, with predicted loss‐of‐function (pLoF) variants accounting for only 3.9% (466 variants; Table S2). However, 77.0% (359/466) of the pLoF variants seen by at least two laboratories were concordantly considered reportable (either P/LP classifications) compared with only 2.6% (149/5,821) of missense variants. While missense variants accounted for a larger fraction of the reportability differences than pLoF variants (75.5%, 77/102; 17.7%, 18/102, respectively), a smaller proportion of total missense variants (1.3%; 77/5821) compared with pLoF variants (3.9%; 18/466) had reportability classification differences (*p* = .0002; Fisher's exact test). The remaining variant types in the data set were synonymous (25.4%; 3009 variants), nonessential splice site intronic (17.0%; 2013 variants), or other (4.6%; 540 variants); however, these had predominantly nonreportable classifications (99.9%, 98.8%, and 99.1%, respectively).

Variants with reportability classification differences (102 variants) were identified in 30 of the 59 genes of interest. To determine trends by disease area, variants in cancer genes and cardiovascular genes from the ACMG secondary findings list were compared. We identified 67 variants with reportability differences in cardiovascular genes, which represents 65.7% (67/102) of all reportability differences and 1.7% (67/3,848) of all shared variants in cardiovascular genes (Table S3). Variants in *LDLR* (17 variants), *MYBPC3* (11 variants), and *MYH7* (9 variants) accounted for the most differences in this disease area (Table S4). Comparatively, 32 variants with reportability classification differences were identified in cancer genes, which represents 31.4% (32/102) of all reportability differences and 0.4% (32/7,954) of all shared variants in cancer genes (Table S3). Variants in *MSH2* (7 variants) and *MLH1* (7 variants) accounted for the most differences in this disease area (Table S4).

### 3.2 Harmonization of reportability differences

To facilitate resolution of the 102 variants with reportability classification differences, participating laboratories first focused on genes of interest with both reportable and nonreportable disorder associations (Figure 1, “Exclusion of non‐reportable disorders”). For example, *RYR1* is associated with autosomal dominant malignant hyperthermia and autosomal recessive myopathies; however, ACMG secondary findings recommends only reporting *RYR1* variants causative for autosomal dominant malignant hyperthermia (Kalia et al., 2017). Within this study variants classified by one laboratory as VUS for the reportable condition, autosomal dominant malignant hyperthermia, and classified by another laboratory as Pathogenic for a nonreportable condition, such as autosomal recessive myopathy, were initially marked as conflicting. However, with this process, eight variants (7.8%) were resolved through consensus that the disorder association is not reportable within the AoURP. These eight variants are marked as “Not Reportable ‐ different disorder” (Table S1) as even though laboratories agree that the variant should not be reported within AoURP the classification differences persist in each laboratories’ internal databases due to the disorder context differences. These “Not Reportable” resolutions are in contrast to variants resolved by reassessment in which all participating laboratories have reclassified the variant.

For the remaining 94 variants with reportability differences, harmonization next focused on reassessment by a single laboratory, primarily focusing on reassessment by the laboratory with the oldest classification (45.7% of remaining differences; 43/94 variants) or the laboratory with an outlier classification (27.7% of remaining differences; 26/94 variants). For example, if three or four laboratories have classified the variant, with two out of three or three out of four laboratories classifying the variant as one class and one laboratory classifying the variant as a different class, the laboratory with the outlier classification would reassess first. For 26.6% of remaining differences (25/94 variants) the classification date(s) were either unavailable for comparison or the classification dates were <1 year apart and reassessment assignments were arbitrarily chosen. With these assignments, reassessment with current guidelines and available evidence by a single laboratory resolved 42.6% (40/94 variants; Figure 1, “Round 1”). One risk allele (p.Ile1307Lys in *APC*) was identified in this first round of reassessments and laboratories agreed to resolve as Not Reportable given the low penetrance of the variant. For variants that remained unresolved after initial single laboratory reassessment (54 variants), the laboratory that performed the reassessment would provide their rationale and evidence and then the other laboratory(ies) would reassess subsequently. This process of reassessment continued until the remaining 54 variants (57.4% of reassessed variants) were resolved (Figure 1, “Round 2”).

For the 54 variants resolved through rounds of reassessment, the majority were resolved by discussion of rationale. Specifically, sharing internal proband data (both proband counts and phenotypes) facilitated resolution for 18.5% (10/54 variants). For 7.4% (4/54 variants), resolution was achieved through consensus regarding mutational mechanisms, specifically loss of function (LoF) variants and the appropriate weight of LoF criteria. Additionally, implementation of gene/disorder‐specific classification guidelines from external expert panels impacted resolution for 33.3% (18/54) of variants.

In total, of the initial 102 variants with differences affecting reportability, 100% were resolved with 56.9% resolved as nonreportable (VUS: 47.1%; not reportable but varying across VUS/LB/B: 9.8%) and 43.1% resolved as reportable (P: 3.9%; LP: 39.2%). By variant type, 44.2% (34/77) of missense variants were resolved as reportable compared with 27.8% (5/18) of pLoF variants that were resolved as reportable (Table S2). By disease area, 56.3% (18/32) of differences in cancer genes were resolved as reportable compared with 38.8% (26/67) of differences in cardiovascular genes that were resolved as reportable (Tables S3 and S4). However, differences by variant type or disease area were not statistically significant.

### 3.3 Harmonization of pathogenic versus likely pathogenic differences

For 0.68% (80 variants) of variants classified by at least two laboratories, the laboratories agreed that the variant met criteria to be reported in AoURP but differed on the classification (i.e., P vs. LP differences). Laboratories prioritized resolution of this conflict type by first focusing on variants that were more frequent, based on internal clinical laboratory case counts or observations in aggregate AoU participant sequencing data. Currently, 55.0% (44 variants) have been resolved, with the majority resolved as P (95.4%; 42 variants) compared with LP (4.5%; 2 variants).

## 4 DISCUSSION

The AoURP aims to create a research resource composed of surveys, biometrics, genetics, and electronic health records from one million participants. Participants can also elect to receive important health‐related information, including an HDR report which will inform individuals regarding clinically significant (P/LP) variants in 59 medically actionable gene‐disorder pairs recommended by ACMG (Kalia et al., 2017). It is anticipated that 2%–4% of AoURP participants will receive an HDR report containing actionable findings (Dewey et al., 2016; eMERGE Clinical Annotation Working Group, 2020). Harmonization of variant classifications within the AoURP is critical to ensure accuracy and consistency in reportable findings. In a pretest launch, the three CVLs and LMM shared all 50,654 unique classified variants in the 59 genes of interest from their internal databases, identifying 11,849 variants with classifications from at least two laboratories. As only variants classified as P and LP will be reported and returned to AoU participants, shared variant classifications were compared to determine reportability concordance. With regard to AoURP variant reportability, classifications were concordant for 99.1% (11,747) of shared variants, with only 0.9% (102 variants) having differences in reportability (i.e. P/LP vs. VUS/LB/B), all of which were resolved after the discrepancy resolution process. By comparison, 3.3% (6,500/194,460) of variants in ClinVar (Landrum et al., 2014) with classifications from ≥2 clinical laboratories had differences of a similar level (P/LP vs. VUS/LB/B); however, the ClinVar data set includes submitted classifications from >600 clinical laboratories, creating more opportunity for discordance, whereas in this study classifications were only compared between four clinical laboratories (ClinVar data from May 1, 2021 accessed from ClinVar Miner (Henrie et al., 2018). Similarly, in the NIH‐funded Electronic Medical Records and Genomics (eMERGE) program, which also focused on reportable findings in the 59 secondary finding genes, the two participating laboratories (BCM and LMM) observed that 2.7% (28/1047) of shared variants had reportability classification differences at the time of comparison in 2016 (eMERGE Consortium, 2019). Since both BCM and LMM harmonized classifications within these genes for the eMERGE program, that may contribute to the lower starting discordance rate within the AoURP.

In this study, the three CVLs and LMM achieved consensus on all 102 variants with reportability differences, with 56.9% resolved as nonreportable in AoURP and 43.1% resolved as reportable. Consensus was achieved by first defining returnable phenotypes in genes with multiple disorder associations, which resolved 7.8% (8/102). The remaining 92.2% of variants (94/102) were resolved by reassessment, with 42.6% (40/94 variants) resolved by an initial reassessment with current guidelines and available evidence. As previous harmonization studies have shown that >50% of variants with discordant classifications are resolved by reassessment with current guidelines and without sharing internal evidence, we chose to only have a single laboratory perform an initial reassessment instead of all participating laboratories immediately sharing internal data and rationale in an effort to minimize reassessment burden (Harrison et al., 2018). However, reassessment with current guidelines and publicly available evidence is not sufficient for complete concordance as classifications between AoURP CVLs and LMM remained discordant for 57.4% (54/94 variants) after initial reassessment with the 2015 ACMG/AMP guideline and accessing the most recent publicly available evidence. This finding is consistent with prior studies that clinical laboratories using the ACMG/AMP guideline can differ in application of criteria, even when presented with the same evidence (Amendola et al., 2016, 2020; Harrison et al., 2017) as well as differ due to the lack of access to internal laboratory evidence (Harrison et al., 2017).

Given the broad scope of the ACMG/AMP guideline for sequence variant classification, specification of evidence types for genes or disorders of interest is necessary to achieve greater consistency in variant classification. The NIH‐funded Clinical Genome Resource (ClinGen) consortium was formed in 2013 to develop standards and processes for evaluating genes and genomic variation to enhance clinical validity and utility (Rehm et al., 2015). As a core goal of ClinGen is expert classification of variants, ClinGen has convened Variant Curation Expert Panels (VCEPs) that focus on gene(s) and create specifications to the ACMG/AMP guidelines for gene‐disorder dyads (Rivera‐Muñoz et al., 2018). Currently, there are five genes on the ACMG secondary finding gene list with ACMG/AMP guideline specifications from ClinGen VCEPs: *LDLR* (Chora et al., 2021), *MYH7* (Kelly et al., 2018), *PTEN* (Mester et al., 2018), *RYR1* (Johnston et al., 2021), and *TP53* (Fortuno et al., 2021). Of the 54 variants with reportable classification differences in this study that were resolved through discussion and multiple rounds of reassessment, implementation of these gene/disorder‐specific classification guidelines from VCEPs impacted resolution for 33.3% (18 variants). For instance, a piece of evidence supporting pathogenicity in the ACMG/AMP framework is “Located in a mutational hot spot and/or critical and well‐established functional domain (e.g., active site of an enzyme) without benign variation,” which is given a Moderate strength level. Application of this line of evidence can be subjective as there is no definitive source of well‐established functional domains, making it difficult to come to consensus when laboratories differ on their definition of a functional domain. Expert defined regions or codons that meet this line of evidence, such as the *MYH7* specifications defining the PM1 applicable region as codons 181‐937 or the *LDLR* specifications selecting 60 highly conserved cysteine residues as qualifying for PM1 criterion, removed the subjectivity in determining when this evidence is applicable and led to concordant reclassifications between laboratories (Chora et al., 2021; Kelly et al., 2018). VCEP specifications will be used throughout AoURP to increase consistency in classifications between CVLs.

In summary, many factors outlined above contributed to the accomplishment of complete concordance with regard to variant classification reportability between the three CVLs and LMM. For one, since AoURP is only returning variants known to cause disorders for which there are established interventions, in accordance with recommendations of ACMG for reporting of secondary findings, we were able to limit the context of variant classifications to the applicable disorders outlined by ACMG. Additionally, given that AoURP funded multiple clinical laboratories to identify and classify variants, harmonization of variant classification was deemed critical to ensure consistency of results being returned to AoURP participants and to prevent individuals with the same variant having different medical management recommendations and follow‐up. Furthermore, complete concordance of reported variant classifications across reporting laboratories is a condition of our FDA IDE protocol. As such, while some laboratories may eventually decide to “agree to disagree,” that was not an option within the AoURP and we were required to come to a consensus classification for all variants. This requirement of harmonization was a significant motivation to harmonize classifications before results are returned to AoURP participants. To facilitate harmonization prelaunch, AoURP formed a Clinical Interpretation and Reporting Working Group, which provided a platform for CVLs to discuss classification rationale and weighting of evidence for variants with reportability differences. Lastly, while prior studies showed that classification differences can persist between clinical laboratories using the ACMG/AMP guideline due to differences in application of criteria, even when presented with the same evidence, these studies focused on harmonization before gene/disorder‐specific classification guidelines from VCEPs were available (Amendola et al., 2016; Harrison et al., 2017). By implementing gene/disorder‐specific classification guidelines, we were able to remove some subjectivity in determining when evidence is applicable and increase concordance between laboratories.

The preharmonization efforts presented here were performed through downloads of a point‐in‐time data set from each laboratory followed by management of work by spreadsheet. Going forward, to ensure ongoing consistency within the real‐time reporting operations of the program, a report harmonization platform (RHP) will be used by all CVLs to generate clinical reports for return to AoURP participants. The RHP will be seeded with each CVL's variant classifications in the 59 genes updated with the outcomes of this harmonization project and regularly refreshed from each CVL's internal knowledge base which supports ongoing clinical testing outside of the AoURP. Data file downloads from the RHP will be available to each CVL so that classifications from other CVLs can be used as an annotation during variant identification and classification, allowing classification differences to be identified early in the workflow. By housing these data files, the RHP will alert CVLs regarding the existence of any conflicts in variant classification at report generation and a report will not be released until the discrepancy is resolved by the CVLs involved. Variants that are unable to be resolved by interlaboratory discussion and data sharing will be reviewed by the AoU Variant Harmonization Subcommittee, that falls within the AoU Clinical Interpretation and Reporting Working Group, and then further escalation to the appropriate ClinGen Variant Curation Expert Panel if available. If resolution is not achieved, the more conservative classification will be applied (i.e., if the discrepancy is LP vs. P, CVLs will default to the LP classification). Finally, all final reported classifications from all identified variants in the 59 secondary finding genes in AoURP participants will be recorded and used as the default reporting classification for future AoURP participants, subject to updated review at least every 6 months.

We anticipate supporting future classification preharmonization projects with new additions to the content of the HDR report. For instance, the ACMG recently released an updated version 3 of the secondary findings list that has been evaluated for future inclusion in the AoURP return of results pending Institutional Review Board and regulatory review (Miller et al., 2021). Upon release of an updated gene list, participating laboratories will follow a similar classification preharmonization project, focusing on the added genes of interest. Additionally, HDR analysis and reporting currently only includes sequence variants but we anticipate adding structural variants after analytical validation and comparison between genome centers and approval by the FDA through a supplemental IDE application.

The AoURP is a groundbreaking research project that will generate a comprehensive data set from over a million participants in the United States. In addition to the research benefits of creating such a data set, enrolled participants can elect to receive an HDR report, which will report on genetic variants known to cause disorders for which there are established interventions. By sharing variant classifications in secondary findings genes, participating laboratories identified and resolved all differences in variant reportability that have been identified to date.

## CONFLICT OF INTERESTS

The authors declare no additional conflicts of interest beyond their employment affiliation.

## Supporting information

Supporting information.

Supporting information.

Supporting information.

Supporting information.

## Data Availability

The data that supports the findings of this study are available in the supplementary material of this article.
